# A199 ALKAL2/ALK AXIS PROMOTE PERSISTENT VISCERAL PAIN

**DOI:** 10.1093/jcag/gwad061.199

**Published:** 2024-02-14

**Authors:** M Defaye, N Abdullah, K Svendsen, L Soussi, A Dickemann, C Altier

**Affiliations:** Physiology and Pharmacology, University of Calgary, Calgary, AB, Canada; Physiology and Pharmacology, University of Calgary, Calgary, AB, Canada; Physiology and Pharmacology, University of Calgary, Calgary, AB, Canada; Physiology and Pharmacology, University of Calgary, Calgary, AB, Canada; Physiology and Pharmacology, University of Calgary, Calgary, AB, Canada; Physiology and Pharmacology, University of Calgary, Calgary, AB, Canada

## Abstract

**Background:**

Long-lasting changes in neural pain circuits precipitate the transition from acute to chronic pain in patients living with inflammatory bowel diseases (IBDs). While significant improvement in IBD therapy has been made to reduce inflammation, a large subset of patients continues to suffer throughout quiescent phases of the disease. Peripheral and central mechanisms contribute to the transition from acute to chronic pain during clinical remission. In the search of new treatments, we previously found that targeting the anaplastic lymphoma kinase (ALK) receptor could have therapeutic value for persistent pain conditions.

**Aims:**

Here we investigated whether ALKAL2 (ALK ligand) /ALK signaling axis regulates chronic visceral pain.

**Methods:**

We used the post-inflammatory dextran sodium sulfate (DSS) mouse model of colitis as a well-established rodent model of chronic visceral pain, in which animals display increased VHS 5 weeks after DSS administration in the absence of inflammation as described in IBD patients. To assess the role of ALKAL2/ALK signalling in chronic visceral pain, we pharmacologically blocked ALK receptor using clinically available compound Lorlatinib in the postcolitis model. Moreover we genetically depleted ALKAL2 in gut-innervating nociceptors that express the heat sensitive transient receptor potential vanilloid type 1 (TRPV1) channel, using shRNA vectorized in cell type specific Adeno-associated Viruses.

**Results:**

We showed that ALKAL2 expression was significatively enhanced in lumbosacral (LS) dorsal root ganglia (DRG) sensory neurons in the post-colitis model. In naïve mice, ALKAL2 administration induced visceral hypersensitivity in an ALK dependant manner. In addition, depletion of ALKAL2 in TRPV1 nociceptors or blocking ALK with the clinically available compound Lorlatinib, reversed visceral hypersensitivity in the post-colitis model. Accordingly, phosphorylation of ALK receptor occurring at the LS of the spinal dorsal horn was reduced.

**Conclusions:**

Overall, these findings provide novel insights into the mechanisms underlying the transition from acute to chronic visceral pain in IBD. Our work could generate a portfolio of clinically available anti-cancer drugs, including Lorlatinib, as novel pain therapeutics. Repurposed ALK inhibitor could facilitate pain management in in individuals with IBD.

**Funding Agencies:**

CIHR

